# Microbial solutions for climate change require global partnership

**DOI:** 10.1128/mbio.00778-25

**Published:** 2025-04-03

**Authors:** J. T. Lennon, R. Rappuoli, D. E. Bloom, C. Brooke, R. M. Burckhardt, A. D. Dangour, D. Egamberdieva, G. K. Gronvall, T. D. Lawley, R. Morhard, A. Mukhopadhyay, R. S. Peixoto, P. A. Silver, V. Sperandio, L. Y. Stein, N. K. Nguyen

**Affiliations:** 1 Department of Biology, Indiana University 123993, Bloomington, Indiana, USA; 2 Fondazione Biotecnopolo di Siena, Siena, Italy; 3 Department of Global Health and Population, Harvard T. H. Chan School of Public Health 1857 , Boston, Massachusetts, USA; 4 Spark Climate Solutions, Covina, California, USA; 5 American Academy of Microbiology, American Society for Microbiology 11003, Washington, DC, USA; 6 Climate and Health Program, Wellcome Trust 5072, London, England, United Kingdom; 7 Institute of Fundamental and Applied Research, National Research University TIIAME 187934 , Tashkent, Tashkent Province, Uzbekistan; 8 Department of Environmental Health and Engineering, Johns Hopkins Bloomberg School of Public Health 25802 , Baltimore, Maryland, USA; 9 Wellcome Sanger Institute, Wellcome Genome Campus 497772 , Hinxton, England, United Kingdom; 10 Georgetown University School of Foreign Service 145743, Washington, DC, USA; 11 Biological Systems and Engineering Division, Lawrence Berkeley National Laboratory 1666, Berkeley, California, USA; 12 Biological and Environmental Science and Engineering Division, King Abdullah University of Science and Technology 564519 , Thuwal, Makkah Province, Saudi Arabia; 13 Department of Systems Biology, Harvard Medical School 198424 , Boston, Massachusetts, USA; 14 Departments of Medical Microbiology and Immunology, University of Wisconsin-Madison 5228, Madison, Wisconsin, USA; 15 Department of Biological Sciences, University of Alberta 98624, Edmonton, Alberta, Canada; Johns Hopkins Bloomberg School of Public Health, Baltimore, Maryland, USA

**Keywords:** climate change, sustainability, economics, health, energy, methane, biofertilizers

## EDITORIAL

Earth is on track for a temperature rise that may exceed 3°C by the end of the century (1). The consequences of climate change are already evident, with more frequent extreme weather events, the emergence of new infectious diseases, and an increased risk of species extinction. Without urgent and effective intervention, the stability of human societies and ecosystems will be severely threatened. Alarmingly, six of the nine planetary boundaries have already been breached (2).

Despite widespread calls to cut greenhouse gas emissions in half by 2030, global emissions continue to rise, reaching a record high in 2023 (3). Now more than ever, effective and affordable strategies for greenhouse gas mitigation and climate change adaptation are needed. The scientific community must unite to identify, develop, and implement these strategies.

Climate solutions have traditionally relied on non-living, or “inorganic,” technologies, which have been instrumental in reducing our reliance on fossil fuels. While renewable energy sources like solar and wind have become more affordable, infrastructure-heavy technologies, such as nuclear power and hydropower remain costly, requiring substantial upfront investment despite decades of development. Effective mitigation of the climate crisis in the coming years demands innovation in both established and emerging technologies to enhance efficiency and accessibility on a global scale.

Now is the time to expand the scope of climate solutions by more fully embracing the potential of biotechnology. While less widely recognized than traditional mitigation strategies, microbe-based innovations can be cost-effective, be adaptable, and have the potential to be highly impactful (4).

The American Society for Microbiology (ASM) and the International Union of Microbiological Societies (IUMS) recently launched an initiative to identify concrete microbial solutions for climate change (5). The organizations convened a diverse group of experts to evaluate existing microbial technologies with the goal of prioritizing solutions. Selection was based on scientific evidence, economic viability, and ease of implementation for people worldwide. The report outlines key microbial solutions and their potential benefits while also examining their economic impact and biosafety considerations. It emphasizes that effective microbial climate solutions must balance both factors without compromise.

The multi-society project demonstrates that breakthrough progress relies on global collaboration across disciplines and sectors (6). While calls for action and partnership are not new (7–10), translating ideas into tangible outcomes remains a significant challenge. The ASM-IUMS initiative not only responds to these calls but also delivers a concrete framework, providing the scientific community and the world with a strong foundation for the crucial next steps.

This is only the beginning. Just as advancements in engineering, physics, and material sciences propelled the Industrial Revolution in the mid-18th century, breakthroughs in the life sciences are driving a Biological Revolution, which holds the potential not only to mitigate the cumulative impacts of climate change but also to safeguard planetary biodiversity. Aided by advances in large-scale data curation and breakthroughs in artificial intelligence, innovations in microbial technologies are opening a powerful new frontier in the bioeconomy, creating sustainable revenue streams that will drive the next era of economic, environmental, and public health progress. However, realizing this potential requires more than just scientific discovery. To fully harness emerging microbial technologies, we must recognize their transformative power, establish a strong ethical framework to guide their application, and collaborate globally across disciplines and sectors. By doing so, we can unlock the full potential of microbial solutions for the benefit of humanity.
